# Manually pressurized droplet digital PCR chip for rapid SARS-CoV-2 diagnostics

**DOI:** 10.1063/5.0180394

**Published:** 2024-02-27

**Authors:** Pinja Elomaa, Tuomas Ojalehto, Darshan Kumar, Ville Jokinen, Päivi Saavalainen

**Affiliations:** 1 Translational Immunology Research Program and Department of Medical and Clinical Genetics, University of Helsinki, Faculty of Medicine, Haartmaninkatu 8, Helsinki 00290, Finland; 2 Folkhälsan Research Center, Helsinki, Finland; 3 Aidian Oy, Espoo, Finland; 4 Aiforia Technologies Plc, Helsinki, Finland; 5 Department of Chemistry and Materials Science, Aalto University School of Chemical Engineering, Tietotie 3, Espoo 02150, Finland

## Abstract

Droplet digital PCR (ddPCR) is a technique in which PCR reaction is divided into thousands of nanoliter-sized droplets and has proven to be a great tool in virus diagnostics. Compared to the gold standard system quantitative real-time PCR (RT-qPCR), ddPCR functions particularly well when dealing with samples with low template counts, such as viral concentration. This feature makes the technique suitable for early detection of the virus. In this study, a novel portable PDMS ddPCR chip is introduced. The chip functions without external pumps using manual pressurization with a multichannel pipet. The created droplets are monodispersed and form a monolayer on the chip's collection chamber, from where they can be effortlessly imaged. Droplets were analyzed and counted using artificial intelligence. The use of the manually pressurized chip was demonstrated for a SARS-CoV-2 assay, which takes advantage of isothermal strand invasion-based amplification (SIBA) technology, allowing quick and accurate, even point-of-care analysis of the sample. The results demonstrate that SIBA assays can be divided into nanoliter-sized droplets and used as quantitative assays, giving an approximation of the samples' viral count.

## INTRODUCTION

Polymerase chain reaction (PCR) based technologies have revolutionized the early detection of viruses such as respiratory syncytial virus (RSV), Epstein-Barr virus (EBV), and dengue viruses.^1–3^ Currently, the most used PCR technology is quantitative real-time PCR (qRT-PCR). Corman *et al.*^4^ were the first to publish primers and probes for SARS-CoV-2 PCR, making qRT-PCR the gold standard for SARS-CoV-2 testing. The established diagnostic workflow for SARS-CoV-2 was proven to be reliable and exclusive. Altogether, the technique is relatively fast-sensitive and allows multiple patient samples to be tested in a single run. However, qRT-PCR has been shown to produce a significant number of false negative results, especially with samples containing low viral counts.^5,6^

To overcome these false negative results, an alternative method, droplet digital PCR (ddPCR), was developed, and it has been proven to be a more accurate and reliable diagnostic tool compared to qR-PCR.^7–9^ In ddPCR, the sample is divided into thousands of nanoliter-sized droplets, some containing one copy (or more) of a template while other droplets contain none. Droplet formation is generally done using a pumping device. Amplification takes place inside these droplets. After amplification, the number of droplets with a positive fluorescent signal are counted and compared to the number of negative droplets.^10^ The advantage over RT-PCR is that even a single template strand can be detected from the massive pool of total DNA. When a single target strand is amplified in a bulk 20 microliter reaction, the amplification signal gets highly diluted and can be too weak to be detected, whereas, within a 1 nl droplet, the signal is concentrated and can be easily seen.^11,12^

ddPCR has been used with clinical samples, especially in the field of oncology, because of its suitability for analyzing, for example, gene copy-number variations, gene expression, and DNA methylation.^13^ The main limitations of the ddPCR are their slow throughput compared to the bulk qRT-PCR and the limited availability due to the requirement of specialized equipment.^14^ On the other hand, when purchasing all the required equipment, the total cost can be prohibitive.^15^

In recent years, advances in molecular biology research have led to the development of alternative nucleic acid amplification technologies (NAATs) to overcome some of the drawbacks of traditional PCR in diagnostic settings.^16^ Isothermal NAATs operate at a constant temperature (below high PCR temperatures), eliminating the need for thermal cycling and sophisticated instrumentation, making them attractive options for rapid and affordable diagnostic solutions in point-of-care (POC) settings. Strand invasion-based amplification (SIBA) is Aidian's proprietary isothermal nucleic acid amplification technology described first in 2014.^17^ Recently, SIBA was applied to compact and portable CE IVD-marked POC SARS-CoV-2 diagnostic test showing sensitivity similar to RT-qPCR.^18^ In SIBA, target template denaturation is based on recombinase activity: homologous recombination, mediated by a recombinase enzyme, between the target template and invasion oligonucleotide (IO) creates local disruption of target double-stranded DNA (dsDNA), allowing amplification primers to anneal. Utilization of single-stranded binding protein in the reaction chemistry stabilizes the denatured dsDNA and prevents the re-annellation of the separated DNA strands. Enzymatic separation of target duplex DNA strands allows the amplification to be carried out at a constant temperature between 40 and 44 °C. The use of target-specific invasion oligonucleotide makes SIBA highly specific and sensitive. The invasion oligonucleotide (IO) has 3′ end 2′-O-methyl RNA modification to prevent it from acting as a template for DNA polymerase. In addition, amplification primers are short enough, so they cannot be used as recombinase substrates.^17,19^ These properties make SIBA inherently resistant to unspecific amplification and false-positive results. This is why the detection can be intercalating dye (e.g., SYBR Green) based. Since digital droplet microscopy can be extremely sensitive, it is crucial that the utilized amplification method is not prone to unspecific amplification. SIBA SARS-CoV-2 assay was optimized by Aidian for digital droplet SIBA application (ddSIBA) by optimizing the reaction conditions for an optimal fluorescent signal for droplet microscopy. Regarding other well-characterized isothermal NAATs, loop-mediated isothermal amplification (LAMP) and recombinase polymerase amplification (RPA) have been demonstrated to be applicable to digital droplet methods ddLAMP and ddRPA.^20,21^

In this study, a pump-free, small and fast all-in-one ddPCR chip has been developed. Microfluidics can be a great tool in biomedicine since everything can be done quickly and using small sample volumes and, therefore, can be exploited in rapid diagnostics.^22^ The microfluidic chip utilizes ddSIBA, which allows all the steps to be done in one chip, from generating the droplets to analyzing the positive and negative droplets. The chip is pressurized by a novel manual pressurization method using a multichannel pipet. In addition, it is shown how the ddSIBA assay can be used in SARS-CoV-2 detection. Chip was imaged, and results were analyzed using a cloud-based image analysis platform, Aiforia, in which deep convolutional neural networks are used to count the droplets. The Aiforia platform has been shown to be a robust and fast analysis tool for images.^23^

## MATERIALS AND METHODS

### Chip design and master fabrication

**FIG. 1.**
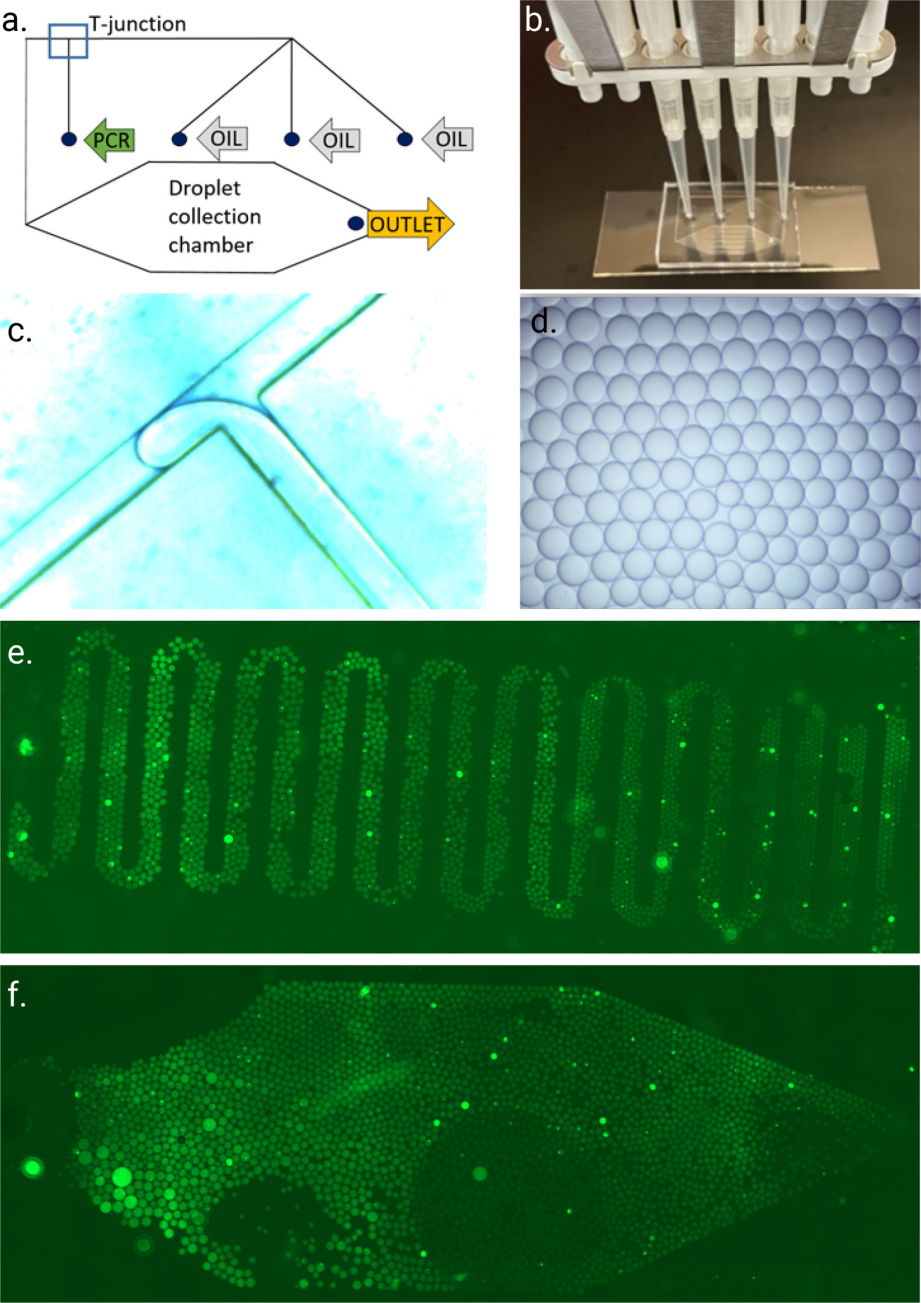
Droplet production on a manually pressurized chip. (a) Schematic picture of the chip, where the green arrow marks the inlet for the reaction mixture, gray arrows mark the inlet for the oils, yellow arrow marks the outlet, and blue square shows the T-junction, (b) pipet and chip, (c) T-junction and droplet formation, (d) uniform droplets, (e) and (f) full chips right after droplet formation: (e) from meander chip and (f) from diamond chip. Figure created with BioRender.

The chip designs were drawn using AutoCad 2020 software (Fig. 1 in the supplementary material) and printed as high-resolution polymer film photomasks (Microlitho). The chip utilizes four inlets (three for the oil and one for the sample), as shown in Figs. 1(a) and 1(b). The 8-channel pipet used for droplet generation gives the user the possibility to influence the droplet size by altering the water-to-oil ratio of the used channels. The inlets lead to a droplet-generating T-junction [Fig. 1(c)], from where the created droplets are brought into a larger collection chamber [Figs. 1(a) and 1(d)]. Two types of collection chambers are utilized: a meander [Fig. 1(e)] and a diamond shape [Fig. 1(f)]. The channel width is 100 *μ*m throughout the chip (except at the collection chambers).

The master for the chips was made using SU-8 photolithography. SU-8 2075 (MicroChem) was spin-coated on top of a silicon wafer at 1250 rpm for 30 s, following soft bake on a hotplate at 65 °C for 5 min and 95 °C for 15 min, UV exposure (Cloe, UV-kub2) through the mask for 10 s, and post-exposure bake for 3.5 min at 65 °C and 9 min at 95 °C. Master was developed by submerging it into SU-8 developer (MicroChem) for 15 min and washing it afterward with isopropyl alcohol. The master was finalized with 15 min of 120 °C hard bake. The channel height used in all chips was measured by profilometry (Bruker Dektak XT).

### PDMS casting and chip finalizing

PDMS (SYLGARD 184, DOW) was mixed with a 10:1 ratio of monomer:crosslinker. A PDMS mixture was degassed and poured on top of the SU-8 master. PDMS curing took place in a 65 °C oven for 2 h. After curing, PDMS was peeled off the master, and chips were cut out. Inlet and outlet holes were bunched at this stage. The chips and glass slides were cleaned using a tape and isopropyl alcohol and dried with compressed air. The chips were bonded on the glass slides with oxygen plasma (plasma oven, Femto Science, 70 ml/min oxygen) 60 W power for 60 s.

### Isothermal SIBA technology

SIBA reactions were carried out using SIBA SARS-CoV-2 assay reagents provided by Aidian Oy. The assay oligos were designed to detect the RNA-dependent RNA-polymerase (RdRp) gene of SARS-CoV-2. Reactions were prepared by combining SIBA A-mix (containing reaction substrates), SIBA B-Mix (containing reaction enzymes), SIBA oligomix (containing 200 nM oligonucleotides and optimized concentration of SYBR Green), nuclease-free water, and SARS-CoV-2 RNA template mixed with sample buffer. SIBA reactions were initiated by the addition of 10 mM magnesium acetate from the sample buffer and incubated at 44 °C for 20–60 min, depending on the experiment.

### Droplet generation and ddSIBA

Droplets were generated using droplet generation oil for Evagreen (Bio-Rad) and the SIBA SARS-CoV-2 reagent (Table I). The template was synthetic SARS-CoV-19 RNA (Exact Diagnostics). The novel innovation was to use an 8-channel pipet to form the droplets: oil from three merging inlet channels and PCR-mix from one [Figs. 1(a) and 1(b)]. Multichannel pipet creates a relatively constant 3:1 flow rate ratio of the two liquids, even when manual pressing itself slightly fluctuates. The best results were achieved using such an 8-channel pipet that guarantees equal pressure to all the tips (Gilson Pipetman), together with 200 *μ*l tips because they create tight sealing in the inlet holes. The T-junction merges the ddSIBA reaction mix into the oil face, forming the water-in-oil droplets. Uniform droplets were collected in the collection chamber as a monolayer to enable their imaging and counting. The uniformity of the droplets was studied using light microscopy (Optika) [example in Fig. 1(d)]. Chips were covered with a thin layer of PMA polymer cover to avoid any evaporation when heating on a hotplate. After the amplification reaction (44 °C 20 min), the chip was ready to be imaged. Some of the droplets are lost during the heating process. Therefore, some figures were taken right after droplet formation (without heating) to count the maximum yield of droplets that can be produced [Figs. 1(e) and 1(f)].

**TABLE I.** ddSIBA 1× reaction mix.

7 *μ*l	SIBA A-mix (substrates)
7 *μ*l	SIBA B-mix (enzymes)
2 *μ*l	SIBA oligomix
2 *μ*l	Nuclease-free water
2 *μ*l	Template RNA in sample buffer

The following section will describe the droplet generation process in more detail. The SIBA mixture was pipetted in the first tube of a tube strip, and droplet generation oil was pipetted into the following three. An 8-channel pipet, with 200 *μ*l tips, was used to draw the liquid into the tips (typically, 20–25 *μ*l liquid to each tip). The tips were carefully placed into the chip inlets—SIBA liquid to the lagging face inlet and the three oils to the continuous face inlets. The tips were pushed almost to the bottom of the chip to ensure tight sealing. At this stage, the chip is prefilled with oil, and, therefore, an empty pipet tip was placed in the outlet hole to collect all the excess liquid. A typical pipet has two stopping points: one that leaves a tiny amount of liquid on the tip and one that empties it. The pipet was slowly pushed to the first stop, simultaneously observing the forming droplets. The liquid stream typically took 5–10 s to stabilize and remove any air from the channels (the air was pushed to the collection chamber). A stream of droplets collecting in the collection chamber was always observed. Typically, near the end of the push, the pipet had to be pushed to the second stop to ensure a continuous flow of the liquids since the flow usually slowed down near the end. The push was stopped when the SIBA mixture or oil in the tips was used, and the tips were carefully lifted from the inlets. This always creates a light backflow of the droplets but settles down quickly. The tip from the outlet hole is carefully removed, and the holes and the collection chamber are covered. The chip is placed in a covered glass Petri dish, and when all the chips are ready, they are transferred to a heating block together. When the chip does not generate even droplets from the start, some maneuvers can be explored, for example, changing the tip angle in the inlet holes.

Evagreen (Biotium 20×) and RNA added to water were used to see the droplets under fluorescence microscopy before heating the system.

### Luna qPCR run

Luna Universal Probe One-Step RT-qPCR was used as a control method for the ddPCR chip. Primers were ordered from IDT as a 2019-nCoV kit: N1 or N2 primers were used. The sample material was synthetic SARS-CoV-2 RNA (Exact Diagnostics). Estimated template concentrations varied between 10 000 and 0.01 (Table II in the Results section). RNA was diluted in water to get the target concentrations. Table III demonstrates the Luna reaction mix and the used PCR cycle. qPCR machine (Bio-Rad) was used for the run.

**TABLE II:** 1× Luna reaction mix and PCR cycle. *means that after the 30 s heating a picture is taken.

10 *μ*l	Luna universal probe one-step reaction mix
1 *μ*l	Luna WarmStart RT enzyme mix
1.5 *μ*l	IDT assay mix (N1 or N2)
2.5–7.5 *μ*l	Water
1–5 *μ*l	Target RNA or DNA
1. 55 °C 10 min	
2. 95 °C 1 min	
3. 95 °C 10 s	
4. 60 °C 30 s *picture	
5. Back to step 3 × 40	

**TABLE III.** Sensitivity of the RT-LUNA and RT-SIBA assays in Figs. 4(a) and 4(b). In RT-Luna, one cycle was 40 s, and in RT-SIBA, one cycle was 30 s.

RT-LUNA		RT-SIBA	
Copies/reaction	Average time to a positive result (min)	Copies/reaction	Average time to a positive result (min)
10 000	13.94	20 000	9.44
1 000	17.15	2 000	11.00
100	19.67	200	13.60
10	21.77	20	25.40
1	23.95	2	N/A
0.1	24.51		

### LAMP

Colorimetric LAMP (Warmstart colorimetric LAMP, NEB) and RT-LAMP isothermal reaction (GspSSD2.0 RT Isothermal Master Mix, OptiGene) were tested alongside the SIBA isothermal reaction. Both LAMP reactions were run at 65 °C for 30 min. Oils used with the LAMP reaction were HFE7500 and FC40 together with 5% fluorosurfactant (Ran Biotechnologies).

### Microscopy and image analysis

Light microscopy images were taken with an Optika light microscope. The ddSIBA reaction mix included SYBRGreen nucleic acid dye and was, therefore, imaged with a fluorescence microscope. Figures were taken with a Zeiss Axio Imager with 5× magnification (BIU Helsinki) using a 38HE GFP filter. The resolution of some of the whole chip figures is low. This is mainly due to the microscope setup: to capture whole chip images, the microscope takes multiple tiny images and stitches them together, forming one large image. Even though the focus level is optimized in several locations throughout the chip, it does not always produce high-quality images (stitching errors and partial unfocused areas).

Image analysis tool Aiforia Create Version 5.5 (Aiforia Technologies Plc, Helsinki, Finland) was used to analyze the fluorescent figures. CNN (convolutional neural network) based AI model was used to detect positive droplets vs background (positive droplets layer) in one AI project and small, normal, and large droplets (droplet size layer) in another AI project. All the AI models were trained using very complex neural depth and set to run for 15 000 iterations (about 4000 iterations were executed). In addition, the AI models were also trained to measure different sizes and intensities using the instance segmentation features. For both AI projects, default image augmentations were used. Total object error was 1.30% and 2.51% for positive droplet and droplet size layers, respectively. Data visualization was done using Aiforia Hub (Aiforia Technologies Plc, Helsinki, Finland), and data analysis was done using Microsoft Excel. From the estimated area of the droplets, the proximate diameter and the droplets' volume could be calculated (Fig. 2 in the supplementary material).

### Real-time detection of SIBA reaction

SIBA SARS-CoV-2 assay was tested using the RT-qPCR instrument in a 384-well plate to ensure the sensitivity of the developed ddSIBA assay. The same reaction mix as with ddSIBA was used with calculated RNA concentrations between 20 000 and 2 RNA copies per reaction. Samples were loaded onto a plate and run with an RT-qPCR instrument (Bio-Rad). RT-qPCR instrument was programmed to conduct 60 cycles at 44 °C for 30 s with SYBR Green reading after each cycle. A melt curve from 30 to 95 °C was performed to assess the SIBA reaction's specificity further.

## RESULTS AND DISCUSSION

### Development of a new technique for ddPCR

The goal of the chip design was to create an easily pressurized system for making droplets that are as monodisperse as possible by utilizing a standard 8-channel pipet for actuating the flow and keeping the ratio of oil vs water flow constant even with a manual push that can slightly vary in rates.

The chip material of choice was PDMS. It is a porous, flexible, and transparent material, which is often used to create microchannel chips due to its malleability. Channel height and width were kept constant throughout the designs (h:150 *μ*m, w:100 *μ*m). The channel height was measured to be 145± 8 *μ*m. The thickness of the chip (PDMS + glass slide) was kept at 2–3 mm. This thickness of the PDMS layer on top of the channels ensures tight sealing of the pipet tips in inlet holes. A thinner chip led to an increased volume of trapped air inside the collection chamber. The microchannel structure leading to the T-junction is the same in all the designs, but the collection chamber varies. Both designs introduced here (diamond and meander) performed equally, and they were optimized to get the maximum filling of the droplets without trapping the air to a particular spot. Nevertheless, the diamond chamber could fit in more droplets per area with a minimal number of air bubbles—it was chosen to be the chip geometry for most of the experiments.

Droplets can be created using any 8-channel pipet, although the best monodispersity level was achieved using a multi-channel pipet that ensures equal pressure to all tips (data not shown). An 8-channel pipet gives the user the possibility to adjust the droplet size by altering the water-to-oil ratio. Different water-to-oil ratios were tested and confirmed that the larger the oil volume compared to the water or PCR mixture, the smaller the droplet size (Fig. 3 in the supplementary material). The aim was to get droplets with a diameter of 100 *μ*m. Therefore, the 3 to 1 ratio was chosen (oil: water). In addition, various channel heights were experimented with. Selected 100 *μ*m channel width will create approximately 110 *μ*m droplets in diameter (Table 1 in the supplementary material).

As PDMS is a porous and gas-permeable material, the evaporation of reagents made standard qPCR assays, and the use of a PCR cycler, difficult. In addition, the transition to isothermal NAAT made the reaction significantly faster and gave us the possibility to develop the chip towards a more portable system. A few different isothermal NAATs were tested to find the most suitable NAAT for the purpose. The most prominent results were observed using SIBA on the chip. LAMP isothermal system was experimented with two different assays: Colorimetric LAMP and RT-LAMP isothermal reaction.^24,25^ Both had unexpected interaction with the used Bio-Rad droplet generation oil: Colori-LAMP showed positive results with a water sample, and when analyzing RT-LAMP (oil included to the mix) using qPCR machine, the signal was significantly weaker compared to the reference sample (data not shown). Other oils (FC40 and HFE7500) were also tested and found to be working moderately. Furthermore, it was observed that the required 65 °C temperature for LAMP was the borderline temperature before the reaction started evaporating during heating. In summary, SIBA was used as the isothermal amplification technology for the developed chip. A list of all the used reagents can be found in Table 2 in the supplementary material.

A time series test was performed to optimize the heating step (Fig. 4 in the supplementary material). The data show that a positive signal starts to develop already at 5 min, and by 20 min, the signal saturates. Some positive droplets were seen after 20 min, but the minimal addition was insignificant. Therefore, a heating time of 20 min was chosen for all further experiments.

### Quality metrics and droplet counts

The total number of counted droplets in the Bio-Rad's ddPCR system must be above a certain threshold to gain a certain sensitivity level and to be considered valid. Even though the aim was to create droplets that are approximately 110 *μ*m in diameter, they were, in reality, slightly larger, around 170 *μ*m in diameter. This was due to the oil flow rate fluctuation—all the three channels were not functional at all times. Therefore, to calculate the number of droplets that can be fitted on a chip’s collection chamber, droplet diameter was assumed to be 200 *μ*m. The total number of droplets that can be fitted to the chip is calculated to be 4510 droplets in meander chips and 5642 droplets in diamond chips. From actual experiments, counted droplets, prior to heating, were 2777 (±20) droplets in the meander and 3000 (±60) droplets in the diamond [Figs. 1(e) and 1(f)]. A portion of the droplets was lost, or they merged during the heating process due to the system's sensitivity.

To assess the quality and repeatability of the assay, all the droplets were counted from three identical runs (after heating). Between the experiments, using a diamond chip, the number of total droplets varied between 1118 and 2124 droplets, from which 37–64 were positive (40 positive droplets expected). In the meander chip, corresponding numbers using 100 copies were 2598−3629 negative ones and 95–115 positive ones (Fig. 5 in the supplementary material). Therefore, in both cases, the number of obtained positives closely matches the number of copies in the sample.

### Monodispersity of the droplets

This study shows how the manually pressurized chip can quickly produce uniform and monodisperse droplets without massive amounts of wasted reagents [Figs. 1(c) and 1(d)].

**FIG. 2.**
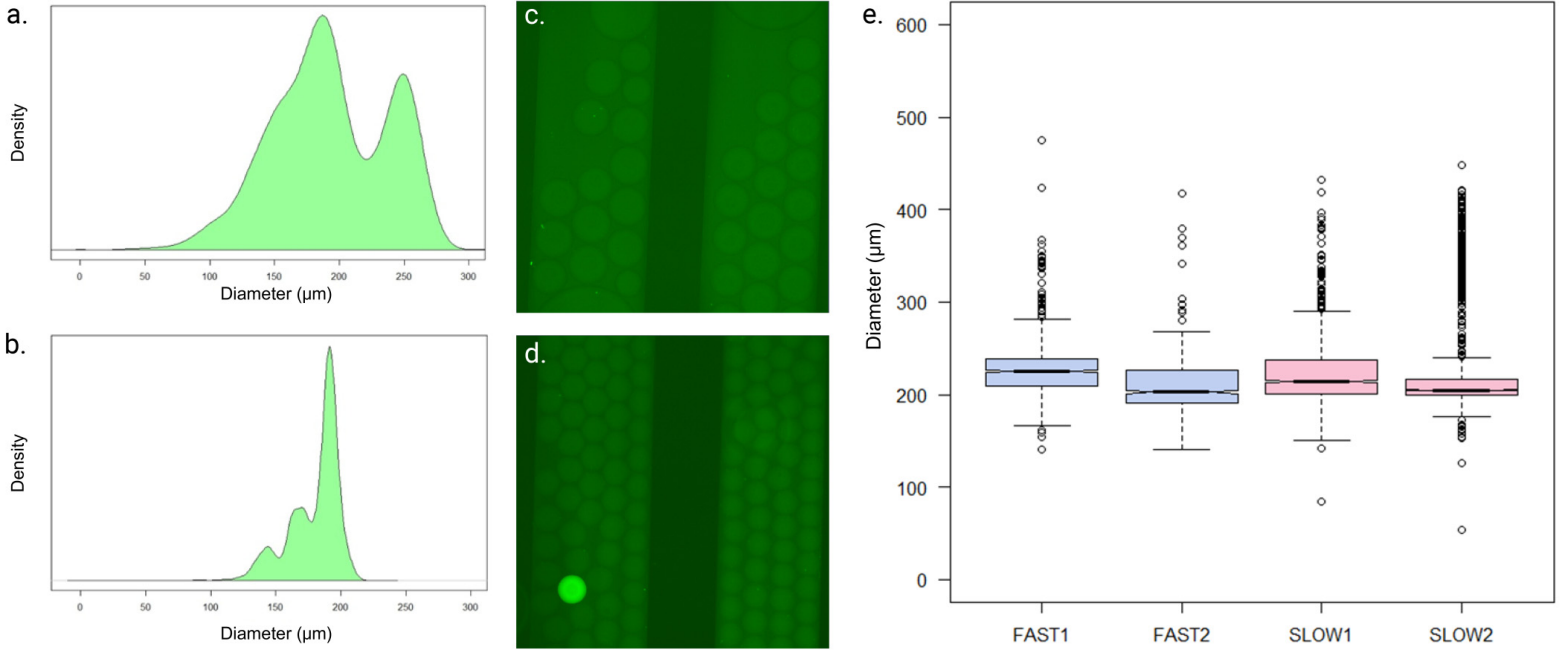
Differing size of the droplets. (a) Diagram of droplet diameters when using an empty chip, (b) diagram of droplet diameters while using prefilled chip, (c) slow push rate droplets, (d) fast flow rate droplets, e.g., a diagram of how different flow rates affected the droplet size: bigger droplets are in the “slow” category, but the overage size does not vary significantly between slow and fast flow rates. Figure created with BioRender.

Two methods were tested for droplet generation to assess the monodispersity; the droplets were either generated directly on an empty chip or alternatively generated on a prefilled chip. The same oil was used in the prefilling step as in droplet generation. The results are shown in Fig. 2; the droplets formed on an empty chip created a two-peaked profile where the droplets were around 180 or 250 *μ*m in diameter [Fig. 2(a)], whereas the prefilled chip created droplets which were approximately 178 *μ*m in diameter [Fig. 2(b)]. Therefore, it can be concluded that it is beneficial to prefill the chip with oil and only then generate the droplets. The likely reason is that filling the chip with oil standardizes the hydraulic resistance at various points of the droplet generation. Although this adds an extra step, the gain in monodispersity is significant.

Monodispersity of the droplets is a key component in ddPCR analytics.^26,27^ The effect of the flow rate (how fast the pipet was pressed) was tested to determine whether it affected the droplet size distribution. A slow flow rate (0.45 *μ*l/s of oil and 0.15 *μ*l/s of sample) was compared to a fast flow rate (0.9 *μ*l/s of oil and 0.3 *μ*l/s of sample). Due to the design, the ratio of the continuous to the discontinuous phase is always 3:1. The droplets were generated in the squeezing mode in both cases [Figs. 1(a) and 1(b)]. The monodispersity of the droplets can be estimated using the polydispersity index (PI), which is based on the size heterogeneity of a sample.^28^ Collected data showed that the press rate did not have a significant effect on either the average size of the droplets or the polydispersity index, although the droplets with the lower press rate were slightly larger [Figs. 2(c)–2(e)]. The polydispersity index varied between 0.11 and 0.21 (mean 0.14) in the high-pressure droplets and between 0.13 and 0.24 (mean 0.14) in the low-pressure droplets. PI values of <0.05 are more commonly associated with monodisperse samples, whereas PI > 0.7 indicates more polydisperse sample.^28^ Generated droplets fall in between these two thresholds. With microsyringe pumps and a T-junction PMMA chip, PI < 0.02 has been shown.^29^ Therefore, it can be concluded that the manually pressurized droplet chip can produce droplets with intermediary monodispersity, and that there is an expected trade-off between the monodispersity and the lower instrumentation barrier. The monodispersity can be somewhat improved by discarding the first created droplets since they tended to be more polydisperse. The same phenomenon could be seen using commercial pumps: it takes a moment to establish a constant flow of equal-sized droplets.

### Limit of detection and sensitivity

**FIG. 3.**
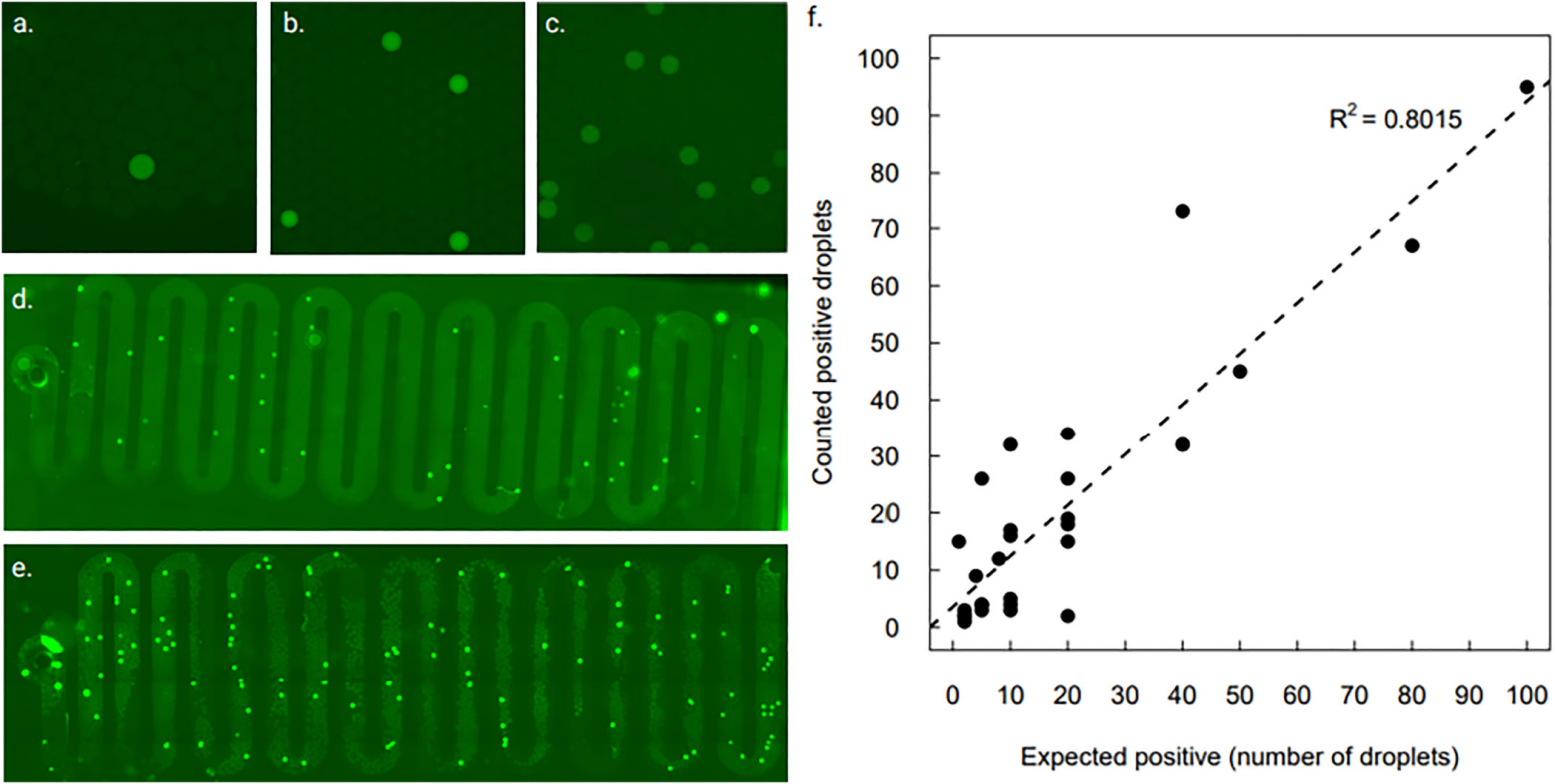
Limit of detection: (a) 1 copy of RNA, (b) 5 copies of RNA, (c) 20 copies of RNA, (d) whole chip image of 50 copies of RNA, (e) whole chip image of 100 copies of RNA. (f) Expected vs counted positive droplets. The graph is based on 17 experiments; two failed experiments are removed from the data shown here. Figure created with BioRender.

A serial dilution of the SARS-CoV-2 RNA was done to determine the limit of detection (LoD) for the ddSIBA chip: RNA concentration was titrated from 200 000 to 0.2 copies of RNA (10^−3^ to 10^−7^) per reaction. RNA was detected up to 2 copies per reaction dilution in all the replicates (n = 5). In Figs. 3(a)–3(c), enlargement figures show different RNA concentrations: in (a), 1 copy; in (b), 5 copies; and in (c), 20 copies of target RNA in the reaction (most of the positive droplets in the close-up figure). The same phenomenon was also observed with higher RNA concentrations in Figs. 3(d) and 3(e) [Fig. 3(d) having 50 copies and Fig. 3(e) having 100 copies of RNA]. Positive droplets were counted from all experiments, and the results were compared to the calculated/expected positive droplet count [Fig. 3(f)]. The best results were seen with small concentrations (1–100 copies per reaction). There is some natural fluctuation in the results since the RNA count in each dilution is only a mathematical estimation of the absolute concentration. In the shown data, it can be seen that the R-squared value 0.80 would indicate that there is a clear correlation between the expected and counted positive droplets.

**FIG. 4.**
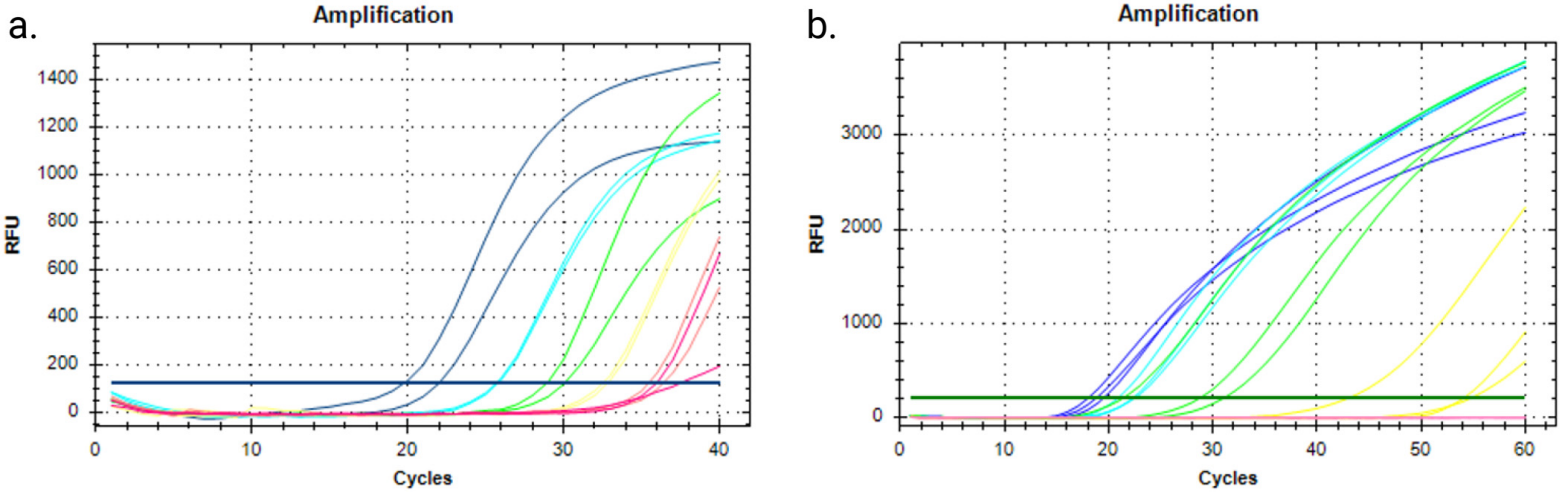
RT-qPCR runs: (a) Luna qPCR reaction with dilution series of the SARS-CoV-2 RNA template, (b) real-time SIBA reaction with dilution series of RNA template. Negative reactions are not shown here, but all the repetitions were negative. Table III shows the average time to get positive results from each concentration. Concentration duplications are shown in the same color. Figure created with BioRender.

RNA dilution series was tested using RT-qPCR Luna assay. Luna was also experimented with the ddPCR chip, but thermal cycling could not be done due to the porosity of the PDMS. The same RNA dilution series was used with SARS-CoV-2 SIBA assay using a qPCR instrument for fluorescence readout. The results indicate how SIBA is comparable with RT-qPCR (Luna) results [Figs. 4(a) and 4(b)].

### Droplet chips without external pumps

There are many pumping devices on the market for droplet generation. While pumps allow highly controllable flow rates, they waste significant reagents in priming the pump/tubings. In addition, they can be time-consuming and laborious. The new system was created keeping it accessible to a large field of people by utilizing tools found in all the laboratories.

The first SARS-CoV-2 ddPCR assay to maintain FDA approval was from Bio-Rad, and it was proven superior in detecting low viral counts.^12^ Stilla introduced to the market droplet Cristal Digital PCR next-generation technology, which showed promising results as a prognostic tool for recognizing SARS-CoV-2 biomarkers.^30^ Soon after the first article, Stilla technology was commercialized into the Naica system.^31^ The Naica system and assay requires up to 80% fewer reagents compared to the normal RT-PCR reaction and is capable of increasing the testing up to tenfold. Stilla has also been harnessed for SARS-CoV-2 detection.^32^

LAMP (loop-mediated isothermal amplification) is one of research's most used isothermal amplification methods.^33,34^ It is a great tool for quick amplification, and it has also been harnessed for use in more quantitative studies. Rane *et al.*^35^ combined the microfluidic PDMS chip and LAMP using flow controllers, and a year after that commercial ddLAMP DropChip was introduced to the markets utilizing centrifuges in droplet creation.^36^ RT-LAMP has also been harnessed to a portable SARS-CoV-2 detection tool with a smartphone-based analysis system.^37^

This study introduces the first-ever ddSIBA application to the public. SIBA has been previously applied to fluorescence readers successfully, and this study demonstrates that it performs exquisitely also in droplet digital applications. The developed assay cannot compete with Bio-Rad ddPCR in droplet counts: the commercial system can produce more than 20 000 droplets per reaction, and the manually pressurized chip around 3000 droplets.^10^ This could be improved in future product development by increasing the droplet collection area and/or decreasing the size of droplets by decreasing the channel width and height. Furthermore, the developed system tops the Bio-Rad system in reagent quantities and time: no reagents go to waste, and the combined time from droplet generation to imaging is less than half an hour compared to the 2–3-h workflow that the Bio-Rad system requires.

In summary, it is demonstrated in the study how the developed ddSIBA system is significantly faster, smaller, and does not require any bulky external pumping devices when compared to Bio-Rad´s ddPCR assay. However, the system cannot compete yet in throughput numbers. These features could make ddSIBA suitable in lower technology environments such as home or field as a point of care test.

### Effectiveness of the new technology and prospects

There have been multiple success stories around pump-free droplet chips taking advantage of, for example, centrifuges or degassing,^26,36,38^ but no existing studies have used a multichannel pipet. Chen *et al.*^39^ utilized a modified single pipet tip to create the droplets. They were able to make monodisperse droplets with the commercial microloading pipet tips, but the droplets were made in a tube instead of a collection chamber, meaning an extra step transferring the droplets to a suitable imagining platform and the whole process takes 1.5 h.

Our chip was designed to be versatile: chips can be used in any wanted ddPCR or ddSIBA assays. Aidian has already developed several SIBA assays for different diagnostic purposes, for example, influenza.^40^ The chip itself has multiple advantages, such as small size, being pump-free, and with a simple interface, which makes it ideal for field laboratories and safety laboratories, to name a few. Due to the lower throughput number compared to the Bio-Rad system, the studied chip functions best with time-sensitive analysis. For example, cases could be surgical samples that must be analyzed during the operation or diagnostics of aggressively spreading pathogens, for example, necrotizing fasciitis bacteria. There are also some fusion genes like the Philadelphia chromosome in leukemia, where the demonstration of physical linkage of fusion genes can be shown by digital droplet assays. Sensitivity and absolute counting of digital assays are also helpful for accurately quantifying gene copy-number variation in some cancers or drug metabolism-related genes affecting the drug efficiency, dosing, and adverse side effects. In all of these examples, early diagnosis is necessary for a chance to have curative or safe treatment. Usually, there are few samples to analyze, but time is in essence, which favors the trade-off for rapidity sacrificing throughput.

**FIG. 5.**
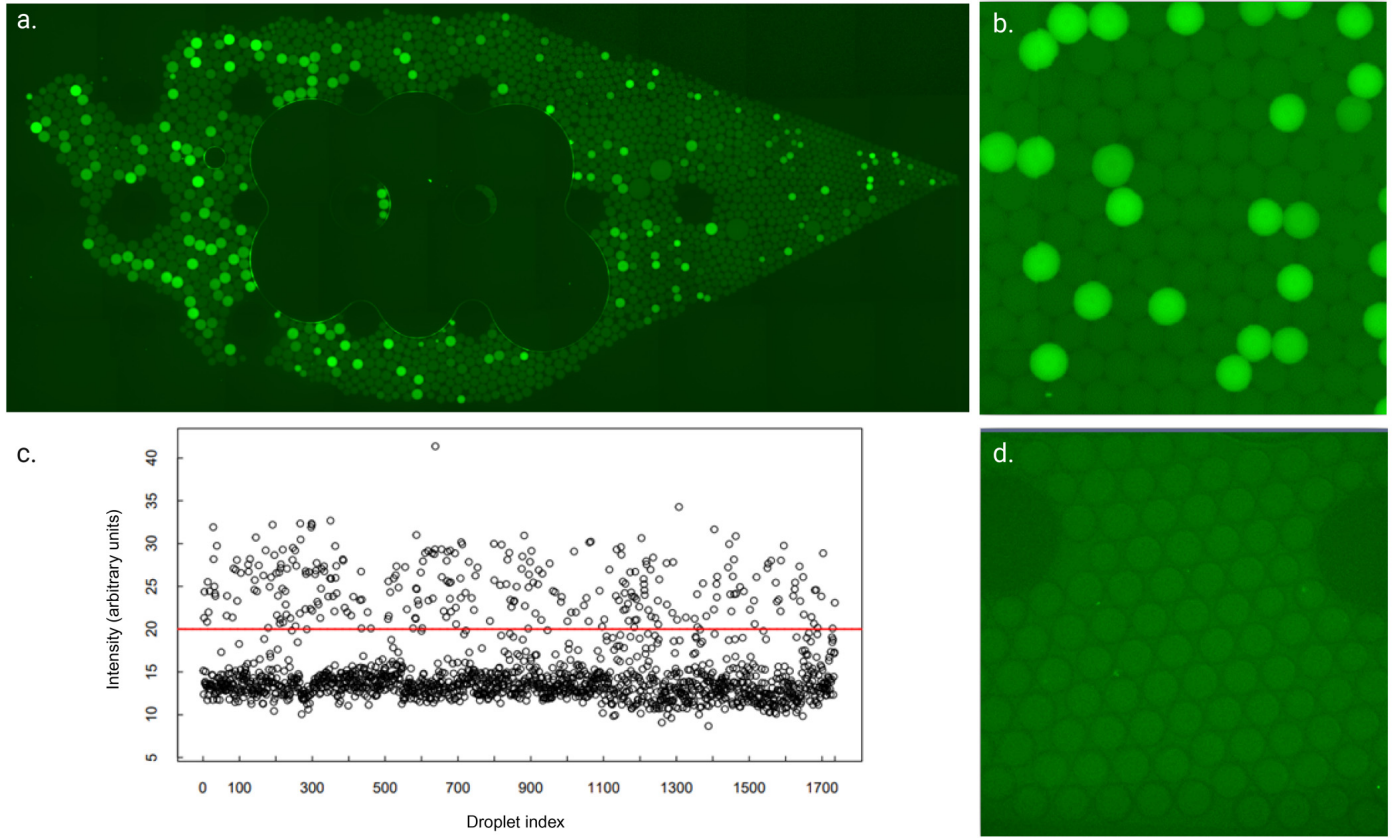
Digital droplet SIBA on manually pressurized chip. (a) Whole chip image illustrating positive and negative droplets and some size differences between the droplets, (b) example of a positive reaction, (c) gray-scale distribution of the positive and negative droplets by Aiforia, (d) example of a negative reaction. Figure created with BioRender.

On the other hand, there are many application possibilities outside of hospital environments, such as veterinary diagnostic-, biosafety-, and field laboratory settings. Small size, portability, and disposability are essential properties for a piece of equipment for the before-mentioned areas. In addition, developing the chip toward single-cell analytics is possible. In addition, possible usage applications are point-of-care (POC) testing. Figure 5(a) showcases a positive sample, including as much RNA as an average SARS-CoV-2 positive patient would in their nasal swab sample.^41^ The difference between the positive and negative samples is clear: droplets containing target RNA, seen here as bright green droplets [Fig. 5(b)], can be detected among the negative, dimmer, droplets [Figs. 5(b) and 5(d)]. This same phenomenon could also be detected with Aiforia AI; two separate groups could be distinguished by their brightness [Fig. 5(c)].

Isothermal NAATs have not been utilized in POC settings as much as PCR. Further research has revealed their reduced specificity due to generating unspecific amplification products, leading to increased levels of false-positive results.^42,43^ SIBA has been demonstrated to be highly specific due to its inherent resistance to unspecific amplification, as described earlier.^17^ SIBA has been recently applied to a POC test in the Egoo Health system by Qlife.^18^ Egoo system performs the SARS-CoV-2 test in 30 min directly from the nasal swab sample. The test is susceptible and will give positive results even as low viral count as 2–4 copies per reaction. It has been shown in this study that the same technology can be used in a droplet-based system with equal sensitivity.

Although the developed system has proven to be functional, some usability aspects should be considered in future mass fabrication. Like many microfluidic systems, the droplet formation is affected by external movement and pressure changes. Due to that, in the future, it would be beneficial to build an optical reader with an integrated heating system to scan the chip immediately after droplet generation and avoid additional chip-moving steps. Another issue was the manual insertion of the pipet tips into punched holes in PDMS; even with the optimized PDMS thickness of 2 mm, some of the oil channels were being incompletely utilized, resulting in variation in the droplet diameter and typically larger droplets in general. This could be alleviated by making a frame that standardizes the pipet tips' insertion depth.

In this study, the aim was to see whether ddSIBA could be quantitative. Similar results have been demonstrated in recently accepted paper.^44^ The results show a correlation between the expected and counted positive droplets and suggest plausibility for ddSIBA being quantitative. Droplet merging was one of the issues that made it difficult to count the absolute positive droplet count since there was no way to find out how many copies of RNA were inside the big droplet from the start. We reduced the amount of merged droplets, but in some experiments, there were multiple positive merged droplets. In addition, low concentrations seemed to work better than higher concentrations, where the observed positive droplet counts were never higher than the expected numbers; this suggests either the inhibitory effect of some RNA template buffer components in higher concentrations or, alternatively, the increasing chance of more than one RNA template within one droplet. In conclusion, more experiments need to be conducted to prove the quantitativity of ddSIBA. The chip could be coated to reduce droplet merging, and channels could made smaller to increase the total droplet count and increase the likelihood that only one copy of RNA goes into a single droplet.

## CONCLUSIONS

Our study successfully demonstrates how a pump-free chip can be utilized for SARS-CoV-2 testing and droplet digital assays in general. The chip can create thousands of droplets rapidly without external equipment, and the whole experiment is done in less than 30 min, giving the user directional information on the viral counts in the sample in a very rapid fashion. The pandemic has already demonstrated that rapid home-, field-, and point-of-care testing will be a significant part of the future’s healthcare, and our chip is showing a novel example of merging the power of digital assay accuracy and sensitivity with the speed of isothermal amplification.

## SUPPLEMENTARY MATERIAL

Some of the findings mentioned in this publication can be found in the supplementary material. The material includes chip master illustration, information on image analysis with Aiforia, and some oil experiments. In addition, there is a time series of SIBA reactions and example figures of the two droplet chip with different viral concentrations using SIBA.

## Data Availability

The data that support the findings of this study are available within the article and its supplementary material.
